# Curved fibers in turbulent channel flow

**DOI:** 10.1140/epje/s10189-026-00609-4

**Published:** 2026-07-20

**Authors:** Darish Jeswin Dhas, Cristian Marchioli

**Affiliations:** Department of Engineering and Architecture, University of Udine, 33100 Udine, Italy

## Abstract

**Abstract:**

We investigate how breaking the geometric axisymmetry of fibers alters their dynamics when suspended in wall turbulence, using intrinsic curvature as a controlled proxy for symmetry loss. The study is based on a series of large-scale direct numerical simulations of turbulent channel flow laden with flexible fibers with varying lengths and intrinsic curvatures. We find that curved fibers tend to accumulate in the near-wall region of the flow. Analyzing their orientation dynamics, we recorded a marked shift in dynamics in comparison to their straight counterparts as they tend to have a preferential alignment even in the near-isotropic region of the flow. They also tend to tumble at larger rates with increasing curvatures. Furthermore, we also find that fiber dynamics become less sensitive to their curvature with increasing length.

**Graphical abstract:**

**Figure:**
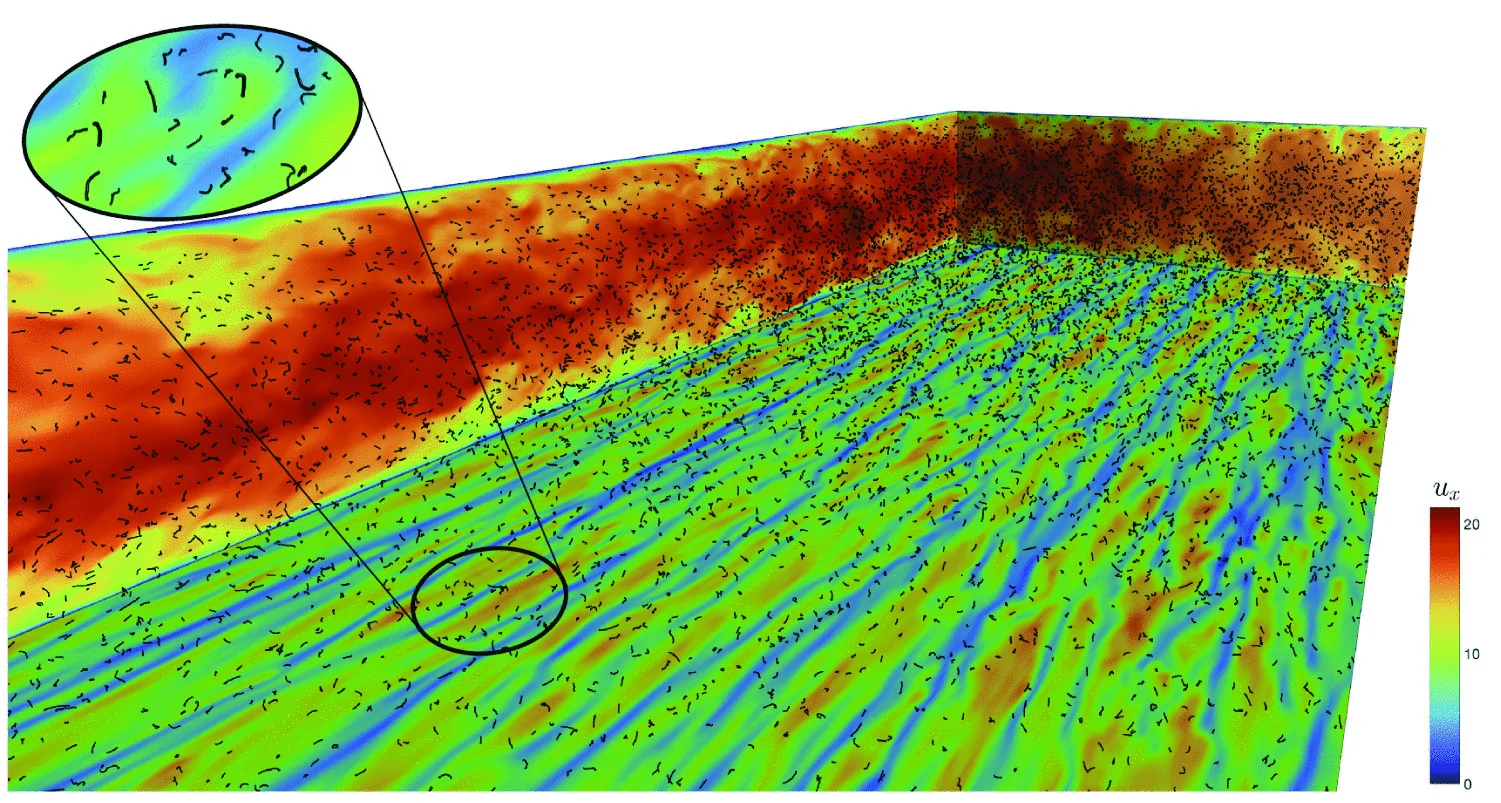

## Introduction

Fiber-laden turbulent flows are widespread in natural and industrial environments, with microplastics representing a prominent example [1, 2]. Substantial evidence indicates that a large fraction of environmental microplastics occurs in fibrous form [3–5]. Consequently, numerous studies in the literature have been devoted towards investigating the dynamics of rigid and flexible fibers in fluid flows [6–24]. However, despite their fibrous form, microplastics often possess geometric or mass irregularities that break their geometric axisymmetry and/or fore–aft symmetry. How do such symmetry breakings modulate their collective behavior when suspended in turbulent flows? In an attempt to address this question, we isolate the effects of geometric axisymmetry breaking on fiber dynamics in wall turbulence by prescribing intrinsic curvature as a controlled proxy for symmetry loss. Such curved fibers feature prominently in microplastic fibers originating from the textile industries [25].

While Jeffery (1922) [26] showed that axisymmetric ellipsoids in simple shear execute a continuous family of closed periodic orbits, later analyses demonstrated how departures from axisymmetry break the degeneracy of Jeffery orbits [27–31]. Under creeping-flow conditions, even weak deviations from axisymmetry cause ellipsoidal particles to undergo doubly periodic motion, characterized by rapid Jeffery rotations coupled with a slow drift induced by symmetry breaking [29]. With the inclusion of weak inertial effects, fluid-inertial torques acting on settling curved fibers align them at oblique angles relative to gravity [31].

Numerical studies show that rigid curved fibers possessing fore–aft symmetry and suspended in simple shear flows undergo cross-streamline drift that depends on their shape and initial orientation, and exhibit chaotic orbits [32, 33]. Cross-streamline drifting dynamics were also seen in asymmetric boomerang shaped particles suspended in a two-dimensional linear shear flow [7, 8, 34]. On the other hand, when otherwise straight fibers deform elastically, their temporally varying shape modifies the corresponding Jeffery orbits through time-dependent orbital constants [35]. These flexible fibers also undergo shape and material-property-dependent lateral migration in Poiseuille flows [36], as they adopt a range of deformation-driven shape configurations even in low-Reynolds-number shear flows [37, 38]. Such deformation-driven drift was also recently seen in elastic hinges suspended in oscillatory shear [39].

To understand the impact of broken geometric symmetries on the dynamics of rigid fibrous suspensions in turbulence, Alipour et al. [40, 41] experimentally investigated long non-axisymmetric fibers in turbulent channel flow. They showed that introducing an intrinsic curvature to rigid fiber suspensions modifies tumbling statistics and promotes wall-normal migration [40]. They also demonstrated that the near-wall dynamics of these fibers are mainly dictated by their curvature irrespective of the shear Reynolds number of the turbulent channel they are suspended in [41]. Subsequent experiments exploited the geometry of mildly curved fibers to reconstruct their full rotational dynamics [42] and to measure the energy dissipation rate of the surrounding turbulent flow [43]. Given the pronounced influence of intrinsic curvature on rigid fibers, a natural question arises: how does their dynamics change when we allow them to deform in the flow? Against this backdrop, we set out to numerically investigate the combined effects of fiber length and curvature on the dynamics of flexible fibers suspended in turbulent channel flows. Towards this, we simulate a dilute suspension of curved fibers, whose density remains comparable to that of the fluid, with the ratio being 1.15. To represent each fiber in our simulations, we connect a series of pointwise elements through a rod-chain formulation [7, 44, 45]. We incorporate a bending torque into the model to control fiber stiffness and an equilibrium angle between the rod elements such that the desired curvature is obtained [44]. We then suspend these fibers in a turbulent channel flow and perform Euler-Lagrange direct numerical simulations of the system.

By evaluating the fiber distribution in the turbulent channel flow, we observe a subtle near-wall accumulation of the fibers, consistent with earlier experimental findings. We also find that the introduction of curvature gives rise to a preferential orientation even in the channel center wherein their straight rigid and flexible fibers counterparts exhibit an isotropic orientation distribution with no discernible bias. In the shear-dominated near-wall region, curvature disrupts the otherwise strong streamwise alignment characteristic of straight fibers. In addition, both increasing fiber length and intrinsic curvature amplify the magnitude of fiber deformation within the channel. An overarching observation is that long fibers progressively lose sensitivity to their intrinsic curvature, as the influence of axisymmetry breaking weakens with increasing length.

This paper is organized as follows. The description of the problem and the governing system of equations is discussed in Sect. 2. The numerical methodology used to solve the governing equations is elucidated in the first part of Sect. 3. Following this, we analyze and discuss fiber distribution in the channel, the orientational dynamics and deformation that the fibers undergo in the channel in Sects. 3.1, 3.2 and 3.3, respectively. Finally, we draw conclusions based on our findings in Sect. 4.

## Problem formulation

We consider a large swarm of curved fibers suspended in a pressure-driven turbulent channel flow. We perform direct numerical simulations that resolve the fluid in the Eulerian frame and track the fibers in the Lagrangian frame. We construct each fiber by connecting a series of rod elements, each smaller than the smallest dynamically relevant flow scale, the Kolmogorov length scale. Using the correlation proposed by Cox [46], we map rods of radius *a* and aspect ratio λ to equivalent ellipsoids with radius $a_{ell} = a \sqrt{\ln {(\lambda )}} / 1.24$. This mapping allows us to model the individual rod elements as point particles and compute their translational and rotational dynamics from the force and torque balance equations for ellipsoids suspended in Stokes flow [26, 47]. We then connect these rod elements by imposing endpoint constraints to assemble long fibers using the rod-chain model introduced by Andric et al. [45]. We subsequently describe the dynamics of each point-wise rod elements that make up the fibers using the system of equations describing the position ($\boldsymbol{p}_n$), orientation ($\boldsymbol{o}_n$), linear velocity ($\boldsymbol{v}_n$) and angular velocity ($\boldsymbol{\omega }_n$) of each rod as [44, 48]

1 $$\begin{aligned}&m_n \frac{d \boldsymbol{v}_n}{d t} = \boldsymbol{F}^D_n + \boldsymbol{X}_{n+1} - \boldsymbol{X}_{n},\end{aligned}$$

2 $$\begin{aligned} \frac{d \boldsymbol{\bar{J} \omega }_n}{d t}&= \boldsymbol{T}_n^D + \boldsymbol{H}_n^D + l \boldsymbol{o}_n \times \left( \boldsymbol{X}_{n+1}\right. \nonumber \\&\quad \left. + \boldsymbol{X}_n \right) + \left( \boldsymbol{Y}_{n+1,b} - \boldsymbol{Y}_{n,b} \right) , \end{aligned}$$

3 $$\begin{aligned}&\frac{d \boldsymbol{p}_n}{d t} = \boldsymbol{v}_n,\end{aligned}$$

4 $$\begin{aligned}&\frac{d \boldsymbol{o}_n}{d t} = \boldsymbol{\omega }_n \times \boldsymbol{o}_n, \end{aligned}$$

where

5 $$\begin{aligned} \boldsymbol{\bar{J}} = \frac{m_n a^2}{12} \left[ (4 \lambda ^2 + 3) (\boldsymbol{I} - \boldsymbol{o}_n \boldsymbol{o}_n^T) + 6 \boldsymbol{o}_n \boldsymbol{o}_n^T \right] \end{aligned}$$

is the inertia tensor of the rod element in the absolute frame of reference, $\boldsymbol{F}_n^D$ is the drag force exerted by the fluid on the rod, $\boldsymbol{T}^D_n$ is the hydrodynamic torque due to the relative spin between fluid and rod and $\boldsymbol{H}^D_n$ is the hydrodynamic torque due to the action of the fluid velocity gradients on the rod. Since each rod is shorter than the Kolmogorov length scale, we can reasonably use expressions derived from Stokes flow theory to model the corresponding drag force and hydrodynamic torques. These, in turn, can be written as [49]

6 $$\begin{aligned}&\boldsymbol{F}_n^D = 6 \pi \lambda a \mu \left[ Y_n^A \boldsymbol{\delta } + \left( X_n^A - Y_n^A \right) \boldsymbol{o}_n \boldsymbol{o}_n^T \right] \left( \boldsymbol{u}_n - \boldsymbol{v}_n \right) , \end{aligned}$$

7 $$\begin{aligned}&\boldsymbol{T}_n^D = 8 \pi \lambda ^3 a^3 \mu \left[ Y_n^C \boldsymbol{\delta } + \left( X_n^C - Y_n^C \right) \boldsymbol{o}_n \boldsymbol{o}_n^T \right] \left( \boldsymbol{\Omega }_n - \boldsymbol{\omega }_n \right) , \end{aligned}$$

8 $$\begin{aligned}&\boldsymbol{H}_n^D = - 8 \pi \lambda ^3 a^3 \mu Y_n^H \left( \boldsymbol{\epsilon o}_n \right) : \left( \boldsymbol{\dot{\gamma }}_n \boldsymbol{o}_n \right) . \end{aligned}$$

Here, μ is the viscosity of the suspending fluid, $\boldsymbol{u}_n$, $\boldsymbol{\Omega }_n$ and $\boldsymbol{\dot{\gamma }}_n$ denote the local fluid velocity, rotation and shear rate at the *n*th rod element’s location, $\boldsymbol{\epsilon }$ is the Levi-Civita third rank tensor, and $X_n^A$, $Y_n^A$, $X_n^C$, $Y_n^C$ and $Y_n^H$ are coefficients which depend on the geometric properties of the rod elements. In the interest of brevity, we refer the readers to [17] for the definition of these coefficients.

We now turn to another key property of our fibers - their curvature. We incorporate this geometric feature by imposing a bending resistance torque that enforces a prescribed equilibrium angle, following [44]

9 $$\begin{aligned} \boldsymbol{Y}_{n,b} = - \frac{\pi E_Y a^3}{8 \lambda } \cos ^{-1}(\boldsymbol{o}_n \cdot \boldsymbol{o}_{eq}) \frac{\boldsymbol{o}_n \times \boldsymbol{o}_{eq}}{| \boldsymbol{o}_n \times \boldsymbol{o}_{eq} |}, \end{aligned}$$

where $E_Y$ denotes the Young’s Modulus of the flexible fiber. Finally, $\boldsymbol{X}$ and $\boldsymbol{Y}$ denote the constraint forces and moments that the connected rod elements exert on each other owing to the no-slip constraint between them, which, in turn, is given by the relation

10 $$\begin{aligned} \boldsymbol{p}_n + l \; \boldsymbol{o}_n - (\boldsymbol{p}_{n+1} + l \; \boldsymbol{o}_{n+1}) = 0. \end{aligned}$$

With the above system of equations, fibers of different lengths are constructed by simply varying the number of rod elements that make up each flexible fiber.

Having described the suspended fibers, we now turn to the fluid flow. We model the carrier fluid as incompressible and Newtonian, driven by a pressure gradient between two smooth, parallel channel walls. For this, we write the Continuity and momentum balance equations as

11 $$\begin{aligned}&\nabla \cdot \boldsymbol{u} = 0, \end{aligned}$$

12 $$\begin{aligned}&\rho \left[ \frac{\partial \boldsymbol{u}}{\partial t} + \boldsymbol{u} \cdot \nabla \boldsymbol{u} \right] = - \nabla P + \mu \nabla ^2 \boldsymbol{u}. \end{aligned}$$

Here, $\boldsymbol{u} = (u_x,u_y,u_z)$ denotes the velocity field, *P* represents the pressure field, and ρ is the density of the carrier fluid. We complement the above equations with no-slip and no-penetration boundary conditions at both the top and bottom walls, $\boldsymbol{u} = 0$, and with periodic boundary conditions in the streamwise and spanwise directions.

We next non-dimensionalize the governing equations using wall units, denoted hereinafter by the superscript “+”. We perform this scaling using the viscous length and time scales, $\mu / (\rho u_{\tau })$ and $\mu / (\rho u_{\tau }^2)$, respectively. The reference velocity $u_{\tau }$ is the friction velocity, defined as $u_{\tau } = \sqrt{\tau _w/\rho }$, where $\tau _w$ denotes the wall shear stress. We subsequently non-dimensionalize the angular velocity as $\omega ^+ = \omega \mu / (\rho u^2_{\tau })$, the fiber density as $\rho ^+ = \rho _p/\rho$, and Young’s modulus as $E_Y^+ = E_Y / (\rho u^2_{\tau })$. Furthermore, we adopt the fiber response time defined by Dotto et al. [18] written for a rigid fiber in its fully straight configuration, and use it to obtain the fiber Stokes number as

13 $$\begin{aligned} \textrm{St}_{\textrm{fiber}}= \frac{2}{9} ( a^+)^{2} \rho ^+ \lambda _{tot} \frac{\log {\left( \lambda _{tot} + \sqrt{\lambda _{tot}^2 - 1} \right) }}{\sqrt{\lambda _{tot}^2 - 1}}. \end{aligned}$$

## Direct numerical simulations of turbulent curved-fiber-laden channel flow

Using the governing equations introduced in the previous section, we now outline the numerical framework we adopt to solve them. We advance the fluid phase using a pseudo-spectral formulation, solving the governing equations in modal space on an Eulerian grid. In this, we evaluate the nonlinear advection term in the Navier–Stokes equations, $\boldsymbol{u} \cdot \nabla \boldsymbol{u}$, in physical space and subsequently transform it to spectral space [50]. We then perform spatial discretization using Fourier expansions in the streamwise and spanwise directions (*x* and *y*), which naturally enforce periodic boundary conditions, while we represent the wall-normal direction (*z*) using Chebyshev polynomials. For temporal integration, we employ a mixed implicit–explicit strategy that combines a second-order Adams–Bashforth scheme for the explicit terms with a second-order Crank–Nicolson treatment of the implicit contributions.

We compute the evolution of the fibers by first interpolating the local fluid velocity and velocity-gradient fields to the positions of the discrete rod elements using fourth-order polynomial interpolation. Because the suspension remains dilute, we neglect hydrodynamic and mechanical interactions among fibers. However, we account for interactions with the confining walls through a purely elastic collision model. Specifically, when a rod element intersects a wall, we reflect it back into the domain based on the position of the element closest to the boundary. We carry out all simulations using a custom in-house GPU-accelerated solver, $surf\_gpu$. Additional details regarding the underlying flow solver can be found in [48].

We perform all simulations for a turbulent channel flow laden with flexible fibers at a shear Reynolds number $\textrm{Re}_{\tau }= 300$. We consider a computational domain of size $L_x \times L_y \times L_z = 4 \pi h \times 2 \pi h \times 2 h$, which corresponds, in physical units, to a water flow (with kinematic viscosity $\nu = 10^{-6} \; m^2/s$) with bulk velocity $u_b = 0.3 \; m/s$ and friction velocity $u_{\tau } = 0.015 \; m/s$ in a channel of dimensions $0.25 \times 0.13 \times 0.04 \; m^3$. In wall units, the domain becomes $4 \pi \textrm{Re}_{\tau }\times 2 \pi \textrm{Re}_{\tau }\times 2 \textrm{Re}_{\tau }$. We then discretize this domain into a $512 \times 256 \times 129$ grid. The total aspect ratio of a fiber therefore becomes $\lambda _{tot} = \lambda \times n_{rods}$, where $n_{rods}$ is the number of rods forming each fiber. We set the radius of every rod element to $a^+ \approx 0.045$ and fix the fiber-to-fluid density ratio to $\rho ^+ = 1.15$. These parameters yield a Stokes number of approximately $\textrm{St}_{\textrm{fiber}}\approx 0.003$ for all cases investigated. We also set the bending stiffness $E_Y^+$ for all the fibers in our simulations to a value of $10^4$. We remark here that such value is much smaller than the one characterizing the experimental fibers, which has been estimated to be $E_Y^+ \approx 10^{10}$. The reason for introducing this difference is twofold. First, the objective of the present study is to examine the interplay between fiber flexibility and intrinsic curvature and their impact on collective dynamics. Second, the fiber model employed - see equations (2) and (9) - becomes increasingly stiff with increasing bending rigidity. This introduces severe numerical stiffness in the governing equations and makes the simulations unfeasible.

In every simulation, we keep the total number of rod elements fixed at $6 \times 10^5$, which results in an overall fiber volume fraction of $\phi _0 = 5 \times 10^{-8}$. As indicated by the governing equations in Sect. 2, the simulations are one-way coupled. Table 1 provides a summary of all the simulations performed. We further note that the lowest curvature considered in this work ($\kappa _0^+ = 0.1$) represents an incremental departure from a straight configuration, allowing us to assess deviations in fiber behavior with increasing curvature.

**Fig. 1:**
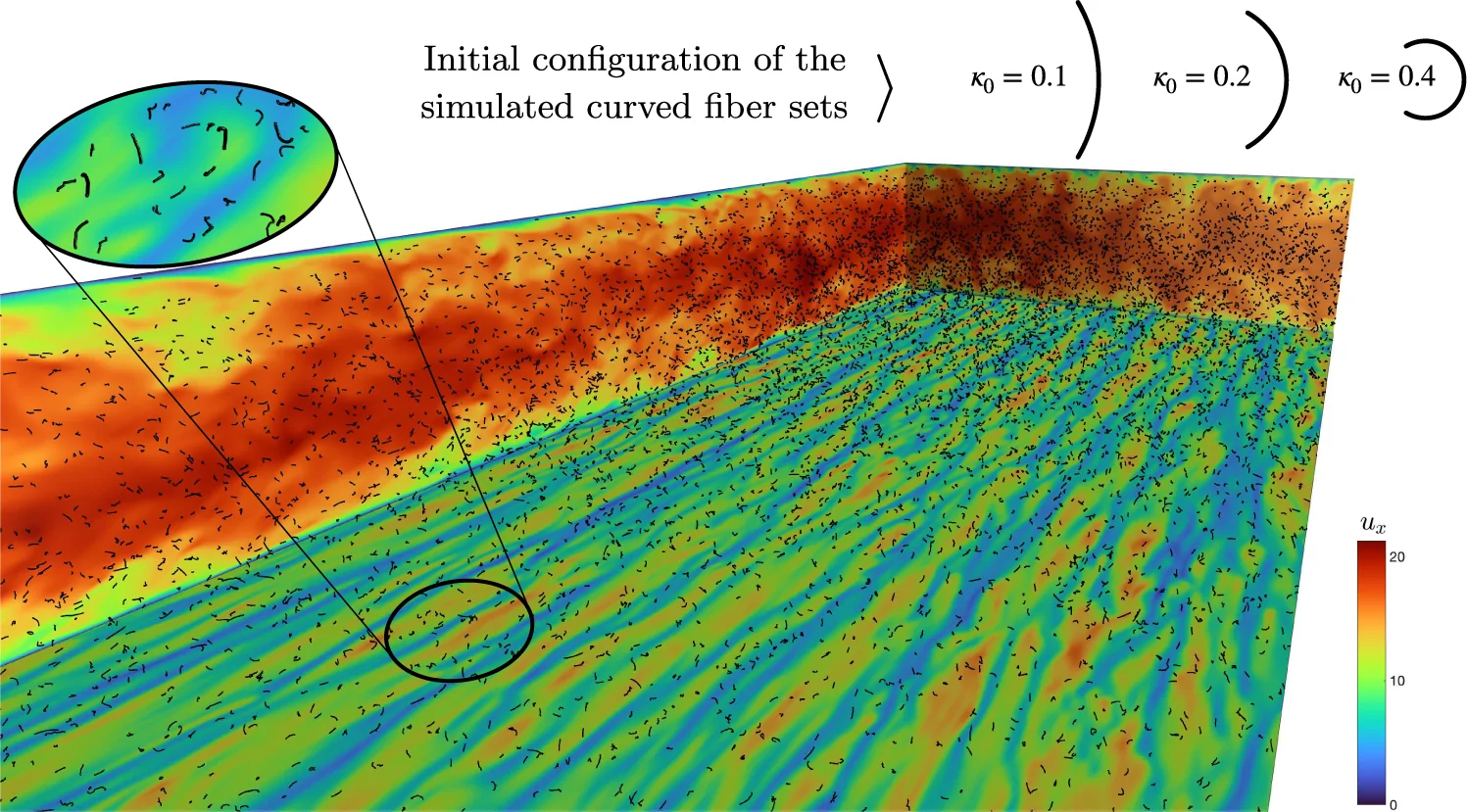
Instantaneous distribution of a collection of curved fibers of length $L_0^+ = 10.5$ and $\kappa _0^+ = 0.4$ in a turbulent channel flow with $\textrm{Re}_{\tau }= 300$. The side and bottom panels display the streamwise component of the velocity $(u_x)$, with the bottom cut at $z^+ = 10$. The fiber thickness is magnified for better visualization. The oval inset highlights fiber deformation near the wall, and on the top right we render the three curvatures $\kappa _0^+$ considered in our simulations

We sample all of our reported statistics at intervals of 15 $t^+$. In evaluating the statistics, we ensure statistical convergence by running every simulation up to 3000 $t^+$ and computing averages solely over a time window of $1500 \le t^+ \le 3000$. In Fig. 1, we display an instantaneous snapshot of the spatial distribution of the longest fibers considered in this study with curvature $\kappa _0^+ = 0.4$. The fiber thickness is exaggerated intentionally for better visualization.

We first describe how we construct the statistical quantities of interest when transitioning from the rod–element representation used in the numerical solver to a fiber-centered description. We characterize the position of each fiber in the channel by its center of mass, which we compute as the arithmetic mean of the positions of all rod elements belonging to that fiber, i.e. $\boldsymbol{p}_{cm}= \sum ^{N}_{i=1}\boldsymbol{p}_i/N$, where subscripts 1 and *N* denote the first and last rod element of each fiber, respectively. We then use the center-of-mass location to assign each fiber to the corresponding wall-normal slab and to compute all fiber-based statistics reported in the following sections, unless specified otherwise.

### Spatial distribution of curved fibers in the channel

**Fig. 2:**
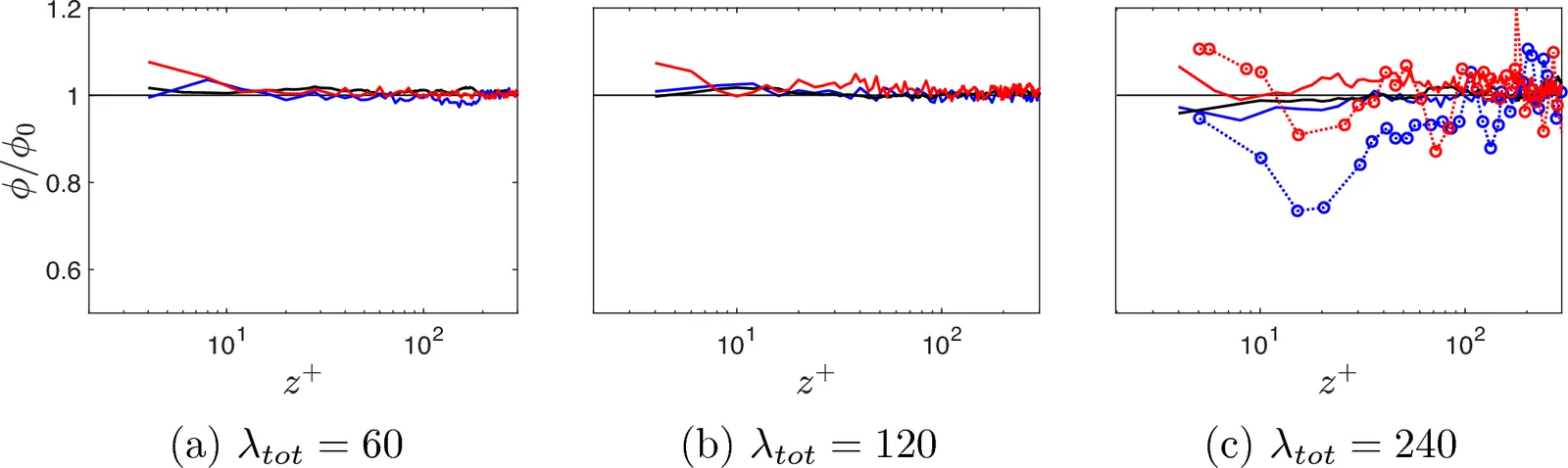
Fiber distribution inside the channel showcased by the normalized volume fraction $\phi /\phi _0$ for fibers of curvatures $\kappa _0^+ = 0.1$ (black), $\kappa _0^+ = 0.2$ (blue) and $\kappa _0^+ = 0.4$ (red), for three fiber lengths corresponding to $\lambda _{tot} = 60$, 120 and 240, respectively. Experimental findings from Alipour et al. [41] on rigid curved fibers of length $L_0^+ = 10.9$ in a turbulent channel flow at $\textrm{Re}_{\tau } = 360$ are shown as ($\cdot \cdot \circ \cdot \cdot$) for curvatures $\kappa _0^+ < 0.28$ (blue) and $\kappa _0^+ > 0.42$ (red)

We begin our analysis by examining how the suspended fibers distribute themselves in the turbulent channel. Towards this, we first compute the local volume fraction (ϕ) of the rod elements within wall-parallel slabs of unit thickness, and subsequently perform a spatial averaging in the homogeneous flow directions and a temporal averaging over the time window $1500 \le t^+ \le 3000$. We then plot the resulting distributions after normalizing them using the initial bulk-averaged fiber volume fraction $\phi _0$. Figure 2 shows the so obtained normalized volume fractions as a function of the wall-normal coordinate, for several combinations of varying fiber lengths and curvatures.

**Table 1:** Parametric combinations of fiber properties considered in our simulations of a turbulent channel flow. Three different fiber lengths, $L_0^+$, and curvatures, $\kappa _0^+$, (values given in wall units) are considered to obtain a total of nine simulations. The entries in the last column are color-coded to indicate the corresponding colors used in the plots for varying curvature unless specified otherwise

	$\lambda _{tot}$	$L^+_0$	$\kappa _0^+$
L1	60	3	0.1, 0.2 & 0.4
L2	120	5.5	0.1, 0.2 & 0.4
L3	240	10.5	0.1, 0.2 & 0.4

A cursory inspection of the volume-fraction distributions reveals a subtle yet systematic effect of fiber curvature across all lengths considered. Regardless of fiber length, introducing intrinsic curvature drives the distribution from an almost uniform profile to a sharply peaked concentration near the wall with increasing magnitude of curvature. Experiments by Alipour et al. [40, 41] have shown the exact same trend emerge with increasing fiber curvature, although those studies consider near-rigid fibers with $E_Y^+ \approx 10^{10}$. Figure 2c also shows a direct comparison between the predictions of our simulations and the experimental findings by Alipour et al. [41] in a turbulent channel flow at $\textrm{Re}_{\tau }= 360$. We attribute the discrepancy in the magnitude of the near-wall peak to the fact that our simulated fibers are not fully rigid and therefore deform under the action of the background flow. This facet is discussed further in Sect. 3.3.

### Orientation and rotation of curved fibers in the channel

**Fig. 3:**
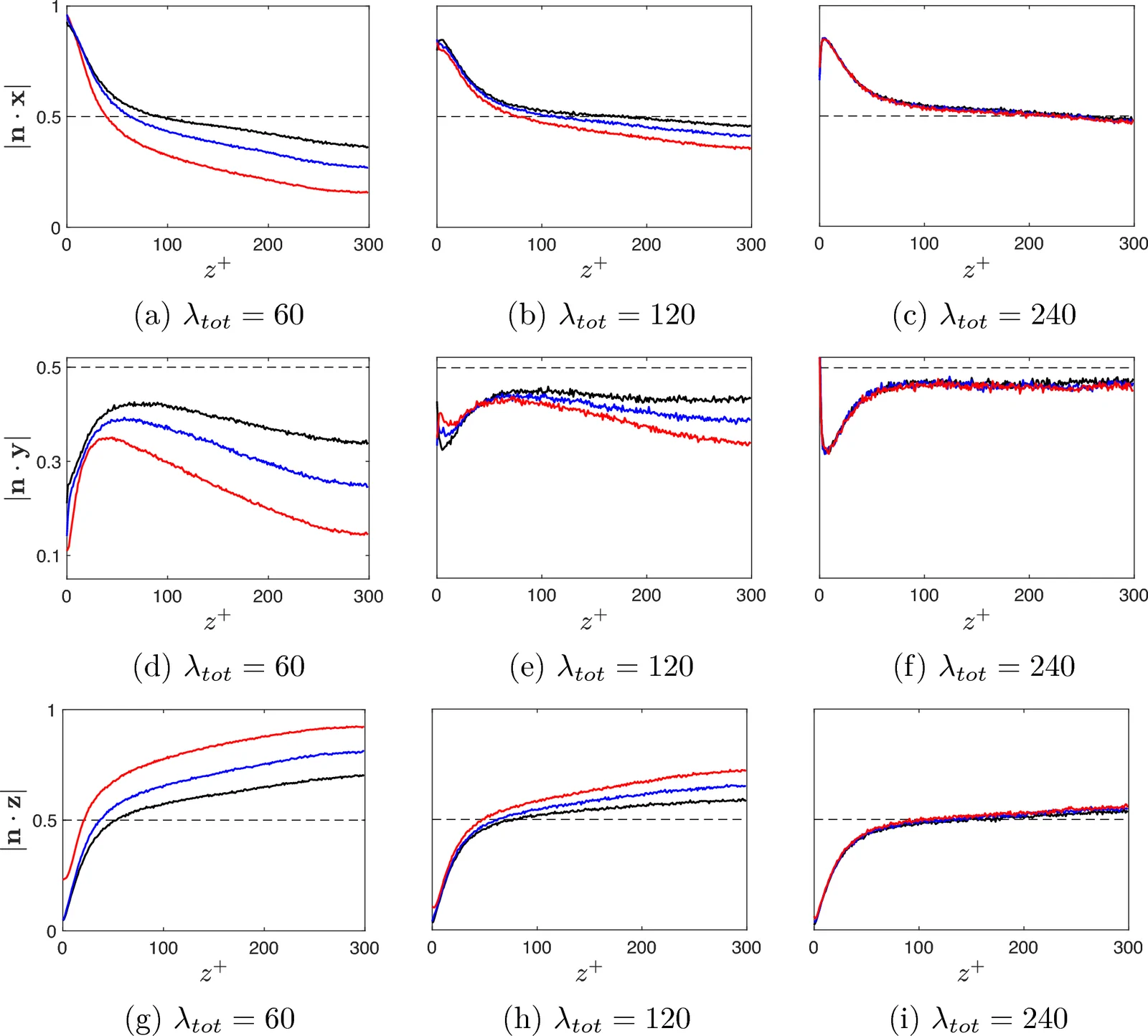
Components of the orientation vector of the fibers with curvatures $\kappa _0^+ = 0.1$ (black), $\kappa _0^+ = 0.2$ (blue) and $\kappa _0^+ = 0.4$ (red), for three fiber lengths corresponding to $\lambda _{tot} = 60$, 120 and 240, respectively

Having probed the distribution of the curved fibers in the flow, we next turn our attention to their orientational dynamics. Towards this, we first evaluate the orientation of the end-to-end distance, an imaginary line connecting the end points of each fiber (referred to as effective distance, hereinafter). Since we have fibers of differing curvatures, we find this technique to remain agnostic to the inherent curvature, unlike computing the normalized mean of the orientation vectors of each rod, which would invariably bias the results based on the fiber curvature. We subsequently condition these orientation values to fibers’ instantaneous wall-normal position in the channel and follow the spatio-temporal averaging described in Sect. 3.1.

Figure 3 shows the components of the obtained orientation vectors for the fibers in our simulations. Numerous studies in the literature show that straight rigid/flexible fibers suspended in the center of a turbulent channel flow exhibit an isotropic orientation distribution, akin to the same when suspended in homogeneous isotropic turbulence [10, 12, 21, 23, 51, 52]. However, the same cannot be said about our simulated curved fibers. Focusing first on the shortest class of curved fibers, we observe a dramatic deviation from the isotropic orientation, indicating a clear bias towards a preferred orientation even in the region of isotropic turbulence. This preferential alignment slowly vanishes with increasing fiber lengths as we observe the longest curved fibers in the channel center of our simulations approach a near-isotropic orientation distribution. As we move away from the channel center, we find that our fibers, irrespective of their length and curvature, exhibit strong alignment with the streamwise direction. This stands in agreement with observations in the literature regarding straight rigid and flexible fibers as the dominant shear in the near-wall region tends to orient the fibers in the direction of the flow [21, 23]. Here again, we observe a notable influence of curvature on the magnitude of the mean alignment with the flow direction, suggesting deviations from the straight-fiber case.

**Fig. 4:**
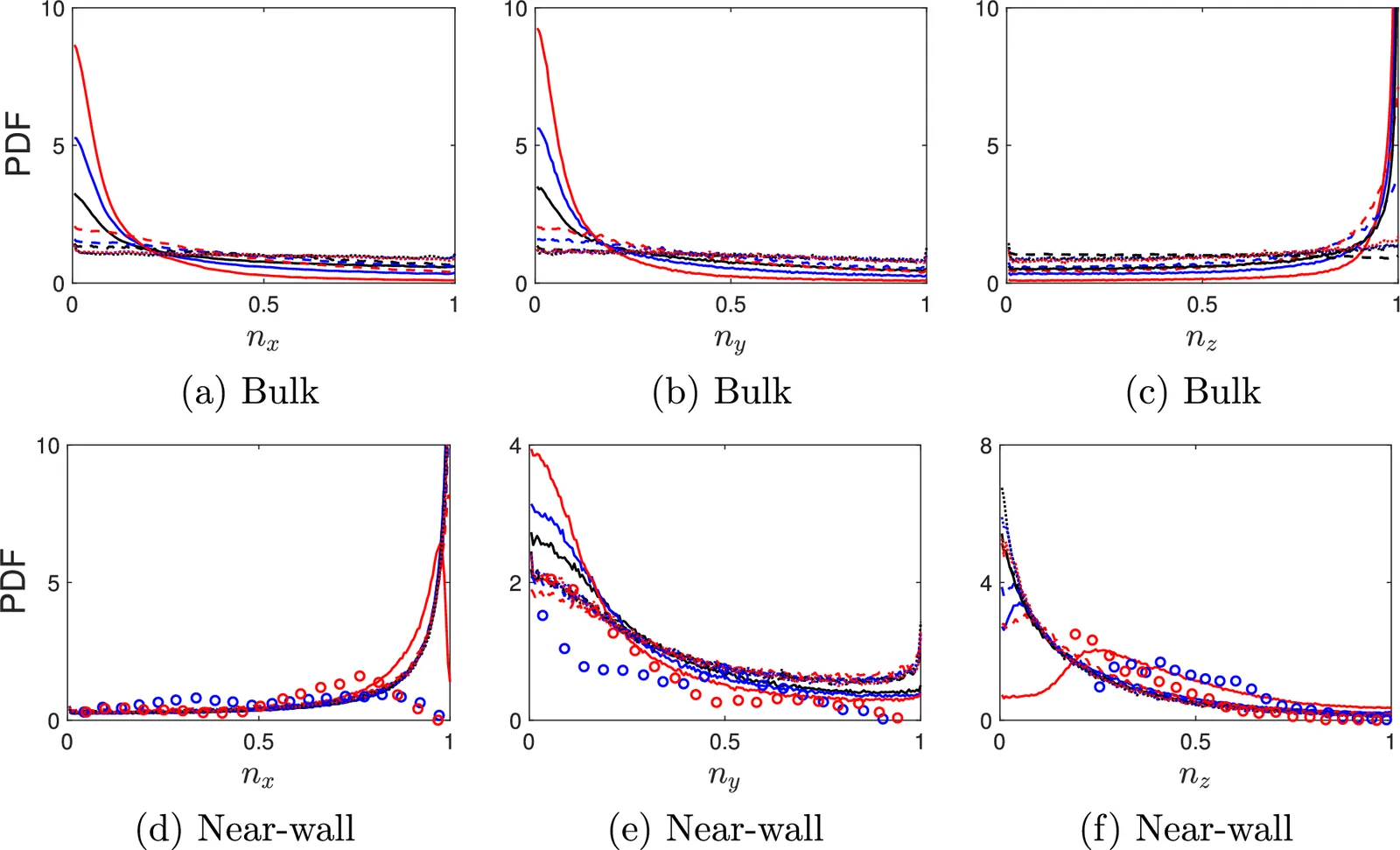
PDFs of the components of the fiber orientation vector with curvatures $\kappa _0^+ = 0.1$ (black), $\kappa _0^+ = 0.2$ (blue) and $\kappa _0^+ = 0.4$ (red), for fiber lengths $\lambda _{tot} = 60$ (——), $\lambda _{tot} = 120$ (---) and $\lambda _{tot} = 240$ ($\cdot \cdot \cdot$). Experimental findings from Alipour et al. [41] on rigid curved fibers of length $L_0^+ = 10.9$ in a turbulent channel flow at $\textrm{Re}_{\tau } = 360$ are shown as (∘) for curvatures $\kappa _0^+ < 0.28$ (blue) and $\kappa _0^+ > 0.42$ (red)

To determine the specific orientation angles preferred by the fibers, we next plot the probability distribution functions of the fiber orientation separately for those located near the channel center, which we refer to as the bulk region - $270 \le z^+ \le 330$, and for those in the near-wall region - $0 \le z^+ \le 30$ and $570 \le z^+ \le 600$, in Fig. 4. Examining the fibers in the bulk region, we find that the shortest curved fibers exhibit strong preferential alignment with the wall-normal direction and anti-alignment with the other two directions. This preferential alignment gradually weakens as fiber length increases, and the distributions approach the isotropic orientation observed for their straight counterparts.

Moving to the probability distribution functions of the orientation of the fibers in the near-wall region reveals markedly different dynamics. As expected, the fibers exhibit the strongest alignment with the streamwise direction, although increasing curvature reduces this bias for the shortest fibers. On the other hand, fiber curvature acts to increase the anti-alignment with the streamwise and spanwise directions, and alignment with the wall-normal direction. These observations indicate that fiber curvature plays a key role in dictating the preferential orientation of the fiber as was also previously noted by Alipour et al. [41]. However, this strong impact gets diminished when looking at the longer fibers in our simulations. Because the fibers in our simulations are not rigid, they frequently deform under the action of the flow, which effectively alters their intrinsic curvature.

**Fig. 5:**
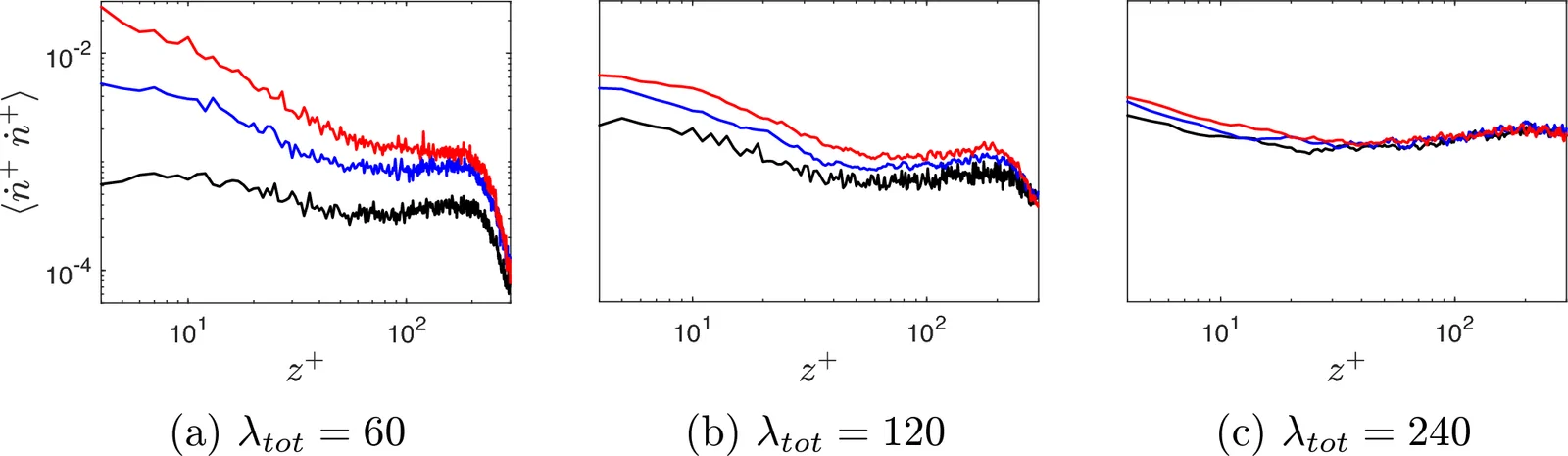
Mean square tumbling rate $\langle \dot{n}^+ \; \dot{n}^+ \rangle$ of fibers with curvatures $\kappa _0^+ = 0.1$ (black), $\kappa _0^+ = 0.2$ (blue) and $\kappa _0^+ = 0.4$ (red), for three fiber lengths corresponding to $\lambda _{tot} = 60$, 120 and 240, respectively

Having documented the dramatic role of fiber curvature on their orientation in the flow, the next question at hand is how this influences their rotational dynamics. To study this, we measure the mean square tumbling rate of the fibers as

14 $$\begin{aligned} \langle \dot{\boldsymbol{n}} \; \dot{\boldsymbol{n}} \rangle = |\omega \times \boldsymbol{n}|^2, \end{aligned}$$

where $\boldsymbol{n}$ denotes the end-to-end orientation vector and ω denotes the mean angular velocity experienced by the rods that make up each fiber. Here again, we evaluate the averaged quantities and plot the mean square tumbling rate as a function of the wall-normal coordinate in Fig. 5. Irrespective of the fibers length and curvature, our fibers in the near-wall region tumble the fastest,as also observed in the case of straight rigid/flexible fibers in the literature [23, 52]. As in the case of orientation, the shortest fibers exhibit the strongest sensitivity to curvature. However, with increase in intrinsic curvature, we record a significant increase in the fibers’ tumbling rate notably in the near-wall region, as can be seen in Fig. 5a for instance. This could be attributed to the fact that with increasing curvature, the near-wall fibers loose their otherwise strong streamwise alignment with the flow, leading to the tumbling rates amplified by the strong shear. Furthermore, the increase in curvature also leads to a decrease in the effective distance, thereby further contributing to the increased tumbling. This magnitude of variation in the tumbling rates decreases with increasing fiber lengths.

### Deformation of curved fibers in the channel

Finally, we explore how fiber curvature influences their deformation dynamics inside the turbulent channel. To evaluate this, we measure the three-dimensional effective (end-to-end) distance of the fibers in the flow, $L_{eff}$. We then normalize this quantity by the corresponding initial effective distance, $L_{eff} (t=0) = L_{eff,0} = 2/\kappa _0^+ \sin {(\kappa _0^+ L_0/2} )$, and apply the same spatio-temporal averaging procedure discussed in Sect. 3.1 to obtain the effective fiber length as a function of wall-normal position. The dimensionless values (in wall units) of $L_{eff,0}$ are shown in Table 2 for the different fiber sets.

**Table 2:** Dimensionless values of the initial effective distance, $L^+_{eff,0}$, for each combination of initial fiber curvature, $\kappa _0^+$, and fiber length, $L_0^+$. The ratio $L_0^+/L_{eff,0}$ is also reported (in parenthesis) and corresponds to the dashed lines in Figs. 6 and 7

$L^+_{eff,0}$ ($L_0^+/L^+_{eff,0}$)	$L^+_0$ = 3	$L^+_0$ = 5.5	$L^+_0$ = 10.5
$\kappa _0^+$ = 0.1	2.989 (1.004)	5.431 (1.013)	10.024 (1.047)
$\kappa _0^+$ = 0.2	2.955 (1.015)	5.227 (1.052)	8.674 (1.210)
$\kappa _0^+$ = 0.4	2.823 (1.063)	4.456 (1.234)	4.316 (2.433)

**Fig. 6:**
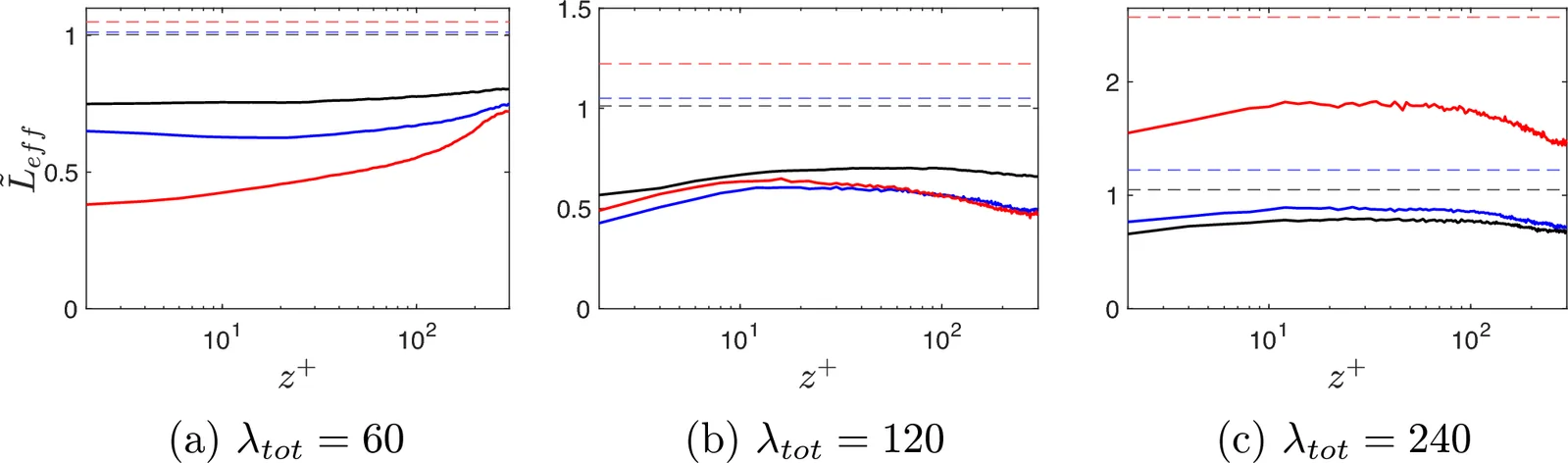
Normalized effective (end-to-end) distance, $\tilde{L}_{eff}$, of fibers with curvature $\kappa _0^+ = 0.1$ (black), $\kappa _0^+ = 0.2$ (blue) and $\kappa _0^+ = 0.4$ (red), for three fiber lengths corresponding to $\lambda _{tot} = 60$, 120 and 240. The dashed lines indicate the maximum attainable value of $\tilde{L}_{\textrm{eff}}$, corresponding to a fully straight fiber. Note that this value depends on the initial fiber curvature, not on fiber length or aspect ratio

In Fig. 6, we plot the resulting dimensionless effective length, $\tilde{L}_{eff}=L_{eff}/L_{eff,0}$, for fibers of varying lengths and curvatures in our simulations. The influence of intrinsic curvature $\kappa _0^+$ on fiber deformation depends strongly on fiber length. For $\lambda _{tot}=60$, increasing $\kappa _0^+$ produces a clear reduction in $\tilde{L}_{eff}$, indicating enhanced deformation. This trend persists for $\lambda _{tot}=120$, although the differences between curvatures diminish. For $\lambda _{tot}=240$, the monotonic ordering breaks down: highly curved fibers instead display larger values of $\tilde{L}_{eff}$ than their less-curved counterparts. This non-trivial reversal can be understood by recognising that intrinsic curvature modifies both the loading experienced by the fiber and the geometric baseline against which deformation is measured. A straight fiber experiences nearly uniform hydrodynamic forcing when aligned with the local flow, and deformation is primarily driven by turbulent fluctuations. Intrinsic curvature, by contrast, introduces geometric asymmetry that can promote non-uniform loading and enhanced deformation. For long fibers, however, the highly curved cases have a much smaller initial effective distance, so they can achieve larger values of $\tilde{L}_{eff}$ even for comparable absolute deformations. The normalised effective length therefore reflects both genuine flow-induced deformation and the geometric constraint imposed by the initial configuration—a distinction that becomes critical for the longest fibers considered here.

**Fig. 7:**
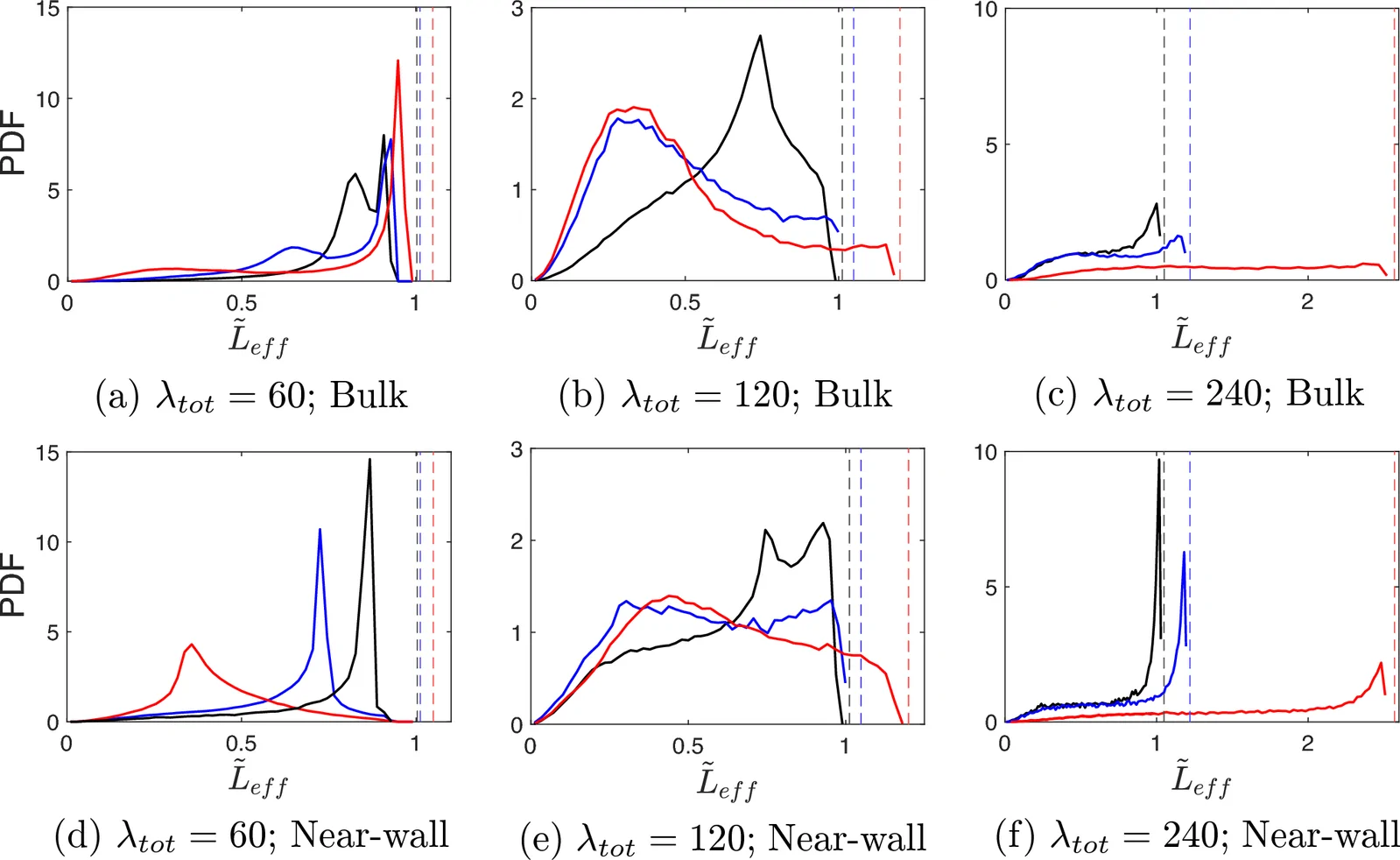
PDFs of the normalized effective (end-to-end) distance $\tilde{L}_{eff}$ of fibers of curvatures $\kappa _0^+ = 0.1$ (black), $\kappa _0^+ = 0.2$ (blue) and $\kappa _0^+ = 0.4$ (red), for three fiber lengths corresponding to $\lambda _{tot} = 60$, 120 and 240. The dashed lines indicate the maximum attainable value of $\tilde{L}_{\textrm{eff}}$, corresponding to a fully straight fiber

The probability density functions of $\tilde{L}_{eff}$, shown in Fig. 7, expose the full distribution of deformation states and reveal features obscured by the mean profiles alone. An additional feature of the distributions is the presence of pronounced peaks close to $\tilde{L}_{eff}=1$, particularly evident for weakly curved fibers and for the longest fibers considered. Since $\tilde{L}_{eff}=1$ corresponds to the equilibrium configuration given by $\kappa _0^+$, these peaks indicate that fibers frequently occupy configurations close to their intrinsic shape despite the action of the turbulent flow. For the shortest fibers, a second peak appears at lower values of $\tilde{L}_{eff}$ and becomes more pronounced with increasing curvature. This bimodal structure indicates that the fibers frequently sample both configurations close to their equilibrium shape and more strongly deformed configurations generated by the turbulent forcing. For $\lambda _{tot}=60$ and 120, increasing $\kappa _0^+$ shifts probability mass toward smaller $\tilde{L}_{eff}$ in both the bulk and near-wall regions, consistent with enhanced deformation. The near-wall distributions are more sharply peaked than their bulk counterparts, reflecting the more constrained configurations imposed by the strong mean shear. For $\lambda _{tot}=240$, the behavior differs qualitatively: the distributions no longer exhibit a simple leftward shift with increasing $\kappa _0^+$. Instead, highly curved fibers display significant probability at larger $\tilde{L}_{eff}$, with extended tails approaching their respective fully stretched limits. As discussed above, this non-monotonic ordering reflects the smaller initial effective distance of highly curved fibers rather than a genuine reduction in flow-induced deformation. These fibers consequently sample a broader range of configurations during their evolution, indicating that their instantaneous shape results from a complex interplay between intrinsic curvature and the local turbulent forcing. Disentangling these two contributions will require either a rigid-fiber baseline at matched curvatures or a decomposition of $\tilde{L}_{eff}$ into geometric and elastic components, which we leave for future work.

## Discussion and conclusions

We investigated the dynamics of fibers with varying intrinsic curvature and length when suspended in wall turbulence, with the specific objective of delineating the role that geometric axisymmetry breaking plays in the system. To this end, we performed direct numerical simulations of flexible fibers constructed using a rod-chain model with a prescribed equilibrium angle to impose the desired curvature, and suspended in a turbulent channel flow at shear Reynolds number $Re_\tau = 300$.

Consistent with experimental findings, we observed a weak accumulation of curved fibers in the near-wall region of the turbulent channel flow. Analysis of their orientational dynamics revealed a substantial change in behavior with increasing fiber curvature. Straight rigid and flexible fibers are well documented to exhibit nearly isotropic orientation distributions near the channel center and strong preferential alignment with the streamwise direction in the near-wall region. Introducing intrinsic curvature, and therefore breaking geometric axisymmetry, fundamentally alters both behaviors. As curvature increases, the fibers progressively deviate from streamwise alignment, in agreement with experiments on curved rigid fibers in turbulent channel flows [40, 41]. At the same time, fibers located in the channel core develop a preferential orientation, with a tendency toward wall-normal alignment. In addition, shorter fibers exhibit significantly enhanced tumbling rates with increasing curvature, particularly in the near-wall region, whereas this sensitivity becomes progressively weaker for longer fibers.

Our simulations differ from the experiments of Alipour et al. [40, 41] in one important aspect, namely fiber rigidity. While the experimental fibers are near-rigid ($E_Y^+ \approx 10^{10}$), the fibers considered here possess a substantially lower bending stiffness ($E_Y^+ = 10^4$). To quantify the consequences of this difference, we examined fiber deformation through the normalized effective (end-to-end) distance. The results indicate that the fibers undergo appreciable deformation, with both increasing contour length and increasing intrinsic curvature amplifying this effect. This difference in rigidity explains why our simulations reproduce the qualitative trends reported in the experiments of Alipour et al. [40, 41] while displaying quantitative discrepancies in the measured statistics. Beyond the individual observations reported here, the results reveal a clear competition between intrinsic geometric asymmetry and flow-induced deformation. Intrinsic curvature introduces a persistent orientational bias that promotes preferential alignment, enhanced tumbling, and increased near-wall accumulation. At the same time, increasing fiber length amplifies deformation and progressively reduces the influence of the equilibrium shape. Consequently, the dynamics of sufficiently long fibers become increasingly insensitive to intrinsic curvature, indicating that flow-induced deformation can partially mask the effects of geometric symmetry breaking.

The present study represents a first step towards numerical investigations of asymmetric fibers in wall turbulence. Several important questions nevertheless remain open. In particular, the mechanism responsible for the preferential orientation observed in the quasi-isotropic core region of the channel remains to be fully understood and will be the subject of future work. Furthermore, the present study has focused exclusively on the effects of axisymmetry breaking. A natural next step is to investigate fibers with fore–aft asymmetry and more complex equilibrium shapes, which are frequently encountered among environmental and anthropogenic fibrous particles. Such studies will help clarify how different forms of geometric symmetry breaking interact with flexibility and turbulence to determine particle transport, orientation, and deformation. The present results provide a foundation for these future investigations by demonstrating that even a simple intrinsic curvature can substantially alter the dynamics of flexible fibers in wall-bounded turbulent flows.
